# The Psychosocial Effects of Taekwondo Training: A Meta-Analysis

**DOI:** 10.3390/ijerph182111427

**Published:** 2021-10-30

**Authors:** Yu-Jin Kim, Seung-Hui Baek, Jong-Beom Park, Sang-Hwan Choi, Jae-Don Lee, Sang-Seok Nam

**Affiliations:** 1 Department of Physical Education, Sejong University, Seoul 05006, Korea; uzkimsp@gmail.com; 2 Department of Health Exercise Management, Sungshin Women’s University, Seoul 02844, Korea; sh100@sungshin.ac.kr; 3 Taekwondo Research Institute of Kukkiwon, Seoul 06130, Korea; jb1907@hanmail.net (J.-B.P.); cshtkd@hanmail.net (S.-H.C.); drlee@kukkiwon.or.kr (J.-D.L.)

**Keywords:** taekwondo, sociality, character, etiquette, school life adjustment, meta-analysis

## Abstract

Taekwondo is a Korean martial art and international sport, and its psychosocial benefits for its trainees have been studied extensively. This review aims to systematically assess and meta-analyze the effects of Taekwondo training on sociality, character, etiquette, and school life adjustment. We searched the RISS, NDSL, and KISS electronic databases between January 1985 and December 2019. We also included gray literature, such as theses, in addition to peer-reviewed articles. R software (version 3.6.2, R Core Team, Vienna, Austria) was used to synthesize the effect sizes and perform moderation analyses. Twenty-eight studies (24 cross-sectional and four intervention studies) were included in the final meta-analysis. Significant positive effects of Taekwondo training were found on sociality (MD = 0.266, 95% CI: 0.191 to 0.341), character (MD = 0.446, 95% CI: 0.331–0.560), etiquette (MD = 0.562, 95% CI: 0.500–0.624), and school life adjustment (MD = 0.308, 95% CI: 0.195–0.421). Overall, the findings of this meta-analysis support that Taekwondo can have a positive impact on the psychosocial factors of trainees. Due to several limitations discussed, well-designed RCTs and multiple levels of Taekwondo intervention studies should be conducted in future research to validate the current findings.

## 1. Introduction

Taekwondo is an internationally established martial art, included in the Olympic Games, and is practiced today by millions of people in more than 200 countries (International Olympic Committee, 2021). Despite some controversies regarding its origin and history, Taekwondo is known to have evolved from a form of unarmed military training of ancient kingdoms of the Korean peninsula, including *Hwarang*, an elite scholar-warrior group of male youth in the Silla kingdom [1]. Though it originated from a fighting system for self-defense, Taekwondo is enjoyed as a sport effective for health promotion and self-discipline, regardless of age and gender. In addition to the physical and psychological benefits of training in a sport, it has also been widely practiced as a vehicle for developing practitioners’ mental strength and ethics [2]. Distinctively, Taekwondo places a significant value in positively affecting practitioners’ behavior and spirit through the training process [3].

The name Taekwondo is derived from the Korean words “tae”, meaning feet or kick, “kwon”, meaning fist or punch, and “do”, meaning path or realization. As its name represents, the Taekwondo spirit can be described by the concept of “do.” This meditative aspect of the forms in Taekwondo includes learning Taekwondo etiquette and the training of minds, encouraging practitioners to achieve self-realization through advancement of both body and mind, and to apply it in their lives [4]. To shape “do,” Taekwondo philosophy teaches five core tenets: courtesy, integrity, perseverance, self-control, and indomitable spirit [5,6]. Historically, these lessons have been associated with pursuing peace, having respect to one another, and standing in solidarity with individuals weaker in body, mind, and spirit [7]. These tenets are consistent with the principle that Taekwondo skills should be used only with good and peace as the ultimate goals. 

One of the reasons behind Taekwondo’s global expansion and popularity over the years may be its positive impact on multiple aspects of personality development such as values, beliefs, self-development, manners, leadership, social skills, and confidence [8]. For decades, studies have reported that it has beneficial effects on trainees’ social development, especially children and adolescents, by improving personality, manners, and behavior, and contributes to adaptation to and satisfaction with school life [9,10,11,12]. 

In terms of personality development, Lim [13] reported that a 12-week Taekwondo intervention for elementary school 2nd-4th graders resulted in significant improvements in leadership, confidence, and manners. Similarly, preschoolers who participated in 15 sessions of a physical activity program using Taekwondo over eight weeks demonstrated higher scores on personality assessments in the lifestyle, self-establishment, sense of community, physical development, and sociality subscales compared with the controls [8].

Taekwondo training has long been reported to be effective in enhancing the social traits of practitioners [3,14,15]. Sociality, a distinct human trait that prompts the need to associate with social groups, is a fundamental characteristic of survival and coexistence with others. According to a study that investigated the correlation between self-regulation and sociality in elementary school students practicing Taekwondo, depending on the duration, the training period explained differences in diligence, interpersonal relationships, and responsibility, suggesting that regular Taekwondo training has a positive effect on social development [16]. They also found that self-regulation affected sociality factors such as diligence, responsibility, interpersonal relationships, and cooperation. Furthermore, in a recent study by Bae and Roh [17], who conducted a 16-week Taekwondo intervention designed for children from multicultural families, it was observed that overall sociability scores improved after the intervention, with reduced scores on feelings of isolation. These findings indicate that individuals learn perseverance and self-control in Taekwondo training, and internalization of these traits may contribute to social development and adaptation to the group. 

Taekwondo has been well received by schools and parents who expected it would assist students in adapting to school life and in forming desirable etiquette and character during a critical period of development. Many of today’s young students are forced to live a uniform and passive life within the framework set by adults, and experience social disconnection due to lack of family integrity, highly competitive education, and school violence, along with increased time spent in online activities [18]. In this context, Taekwondo has been discussed as a possibly effective option for many psychological and social problems, such as the school violence, bullying, and delinquency to which children and adolescents are exposed [19].

Previous studies have provided evidence that Taekwondo can be an effective tool for school life adjustment and the satisfaction of growing children by promoting self-regulation and social development. However, these studies vary in the assessment tools and the subfactors used in their investigations. Furthermore, differences in sample size, sample type, sex, age, and study design (i.e., intervention or cross-section) in past studies also limit the generalizability of the results. Therefore, a systematic integration of studies that have been published to date is required to verify the effects of Taekwondo training on the psychosocial characteristics of practitioners.

This research comprised a meta-analysis of the available literature to provide a systematic review of the effects of Taekwondo training on psychosocial factors including sociality, character, etiquette, and school life adjustment.

## 2. Materials and Methods

### 2.1. Study Selection

This study was performed according to the Preferred Reporting Items for Systematic reviews and Meta-Analyses (PRISMA) statement in the Cochrane protocols [20]. We searched studies conducted between January 1985 and December 2019 using the Research Information Sharing Service (RISS), National Digital Science Library (NDSL), and Korean Studies Information Service System (KISS) electronic databases. The literature search formula was constructed using both Korean and English: ((“sociality” OR “social development”[Title/Abstract])<AND>(“taekwondo”[Title/Abstract])), ((“character”[Title/Abstract]) <AND>(“taekwondo”[Title/Abstract])), ((“manner” OR “courtesy” OR “etiquette” [Title/Abstract])<AND>(“taekwondo”[Title/Abstract])), and ((“school life adjustment” [Title/Abstract])<AND>(“taekwondo”[Title/Abstract])). The search strategy was adapted to each database.

The research papers were first screened according to the population, intervention, comparison, and outcome (PICO) criteria. The participants (P) of our review were limited to Taekwondo practitioners compared with non-practitioner controls. Since this study aimed to examine the effects of Taekwondo training on the general population, studies targeting Taekwondo athletes, demonstration members, Taekwondo majors, instructors, and referees were excluded. The intervention method (I) was a Taekwondo training program, including both intervention and cross-sectional studies. The comparative group (C) comprised those who did not participate in the Taekwondo training. The outcome (O) or dependent variables were sociality (subfactors: activity, autonomy, capability, cooperation, law-abidance, leadership, responsibility, sociability, and stability), character (subfactors: sense of community, confidence, consideration, emotionality, leadership, living, propriety, self-establishment, and self-esteem), etiquette (subfactors: deportment, greeting, interpersonal etiquette, language, listening, phone etiquette, etiquette in public places, dining etiquette, and visiting etiquette), and school life adjustment (subfactors: learning, school events, friendship, rule compliance, and teacher relations). We only included studies that presented the mean and standard deviation of the outcome measures. To reduce publication bias, gray literature, such as theses, was also included in addition to peer-reviewed literature published in academic journals. The languages of the literature were limited to Korean and English.

### 2.2. Statistical Methods

We examined the moderator effects of sample size, sex, age, study type, publication type, and quality of the study on outcome measures. Sex was classified into three categories according to the proportion of male participants. Studies where the proportion of males was 33% or less of the total participants were set as “1,” studies with less than 66% male participants were set as “2,” and studies with 66% males or more were set as “3.” In terms of age, “1” indicated studies of elementary school students or younger, “2” for middle and high school students, and “3” for college students.

We used the Newcastle Ottawa Scale (NOS) recommended by the Cochrane Collaboration, to assess the risk of bias in case-control studies. The NOS contains eight items across the three domains. The selection domain includes four items: (1) adequacy of case definition, (2) representativeness of the cases, (3) selection of controls, and (4) definition of controls. The comparability domain contains one item: comparability of cases and controls on the basis of the design or analysis. The exposure domain has three items: (1) ascertainment of exposure, (2) same method of ascertainment for cases and controls, and (3) non-response rate. A star system was employed to assess the quality of the included studies, with the highest quality in each item receiving a maximum of one star, except for the item in the comparability domain, which allowed the assignment of two stars. The total quality scores evaluated using the NOS ranged from zero to nine stars.

The heterogeneity of the effect sizes was assessed using R version 3.6.2 (The R Foundation, Vienna, Austria). Analysis of the overall effect size was performed when there were three or more identical outcome variables. Since the outcome measures were on five-point scales, all analysis results were presented with mean difference (MD) and 95% confidence intervals (Cl). A random-effects model was used under the assumption that the effects estimated in different studies were heterogeneous [21]. We performed meta-ANOVA and meta-regression analysis for each moderator variable and used a restricted maximum likelihood (REML) to estimate both continuous (e.g., sample size) and categorical variables (sex, age, research period, publication type). Heterogeneity was assessed using the Cochrane’s Q and Higgins I^2^ statistics, with the *p*-value of the Cochrane’s Q test 0.1 or less or the Higgins I^2^ 50% or more judged as statistically significant heterogeneity. We presented publication bias with a contour funnel plot and performed a sensitivity analysis using trim-and-fill, if necessary.

## 3. Results

### 3.1. Characteristics of Included Studies

**Figure 1:**
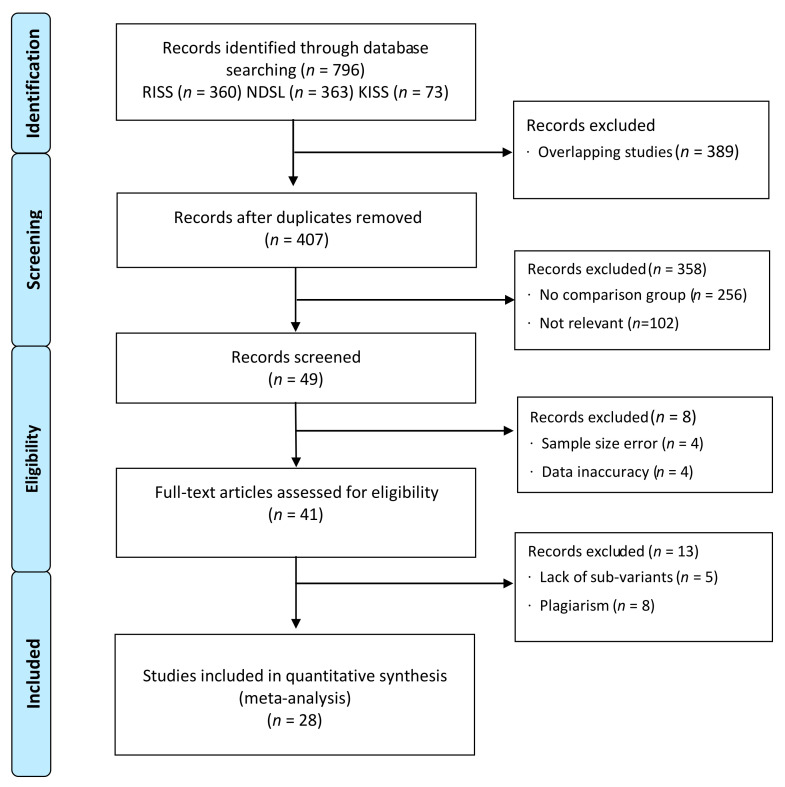
Flow chart of article screening process.

A total of 796 studies (RISS, *n* = 360; NDSL, *n* = 363; KISS, *n* = 73) emerged as a result of the initial database search. Among the searched studies, 768 studies were excluded due to the following reasons: (a) overlapping (*n* = 389), (b) irrelevant to “sociality,” “character,” “etiquette,” or “adjustment to school life” (*n* = 256), (c) no comparison group (*n* = 102), (d) sample size error, (e) inaccurate data analysis (*n* = 4), plagiarism (*n* = 8), and lack of sub-variables (*n* = 5). After article screening, 28 studies fulfilled the inclusion criteria and were included in the final analysis. The PRISMA flow chart of the article screening process is shown in Figure 1. Two independent researchers evaluated the risk of bias according to the NOS criteria of the Cochrane Collaboration. According to the quality evaluation results, 15 of the selected studies had good quality, seven were fair, and six had poor quality. The characteristics of each study are presented in Table 1.

### 3.2. Outcome Measures

#### 3.2.1. Sociality

**Figure 2:**
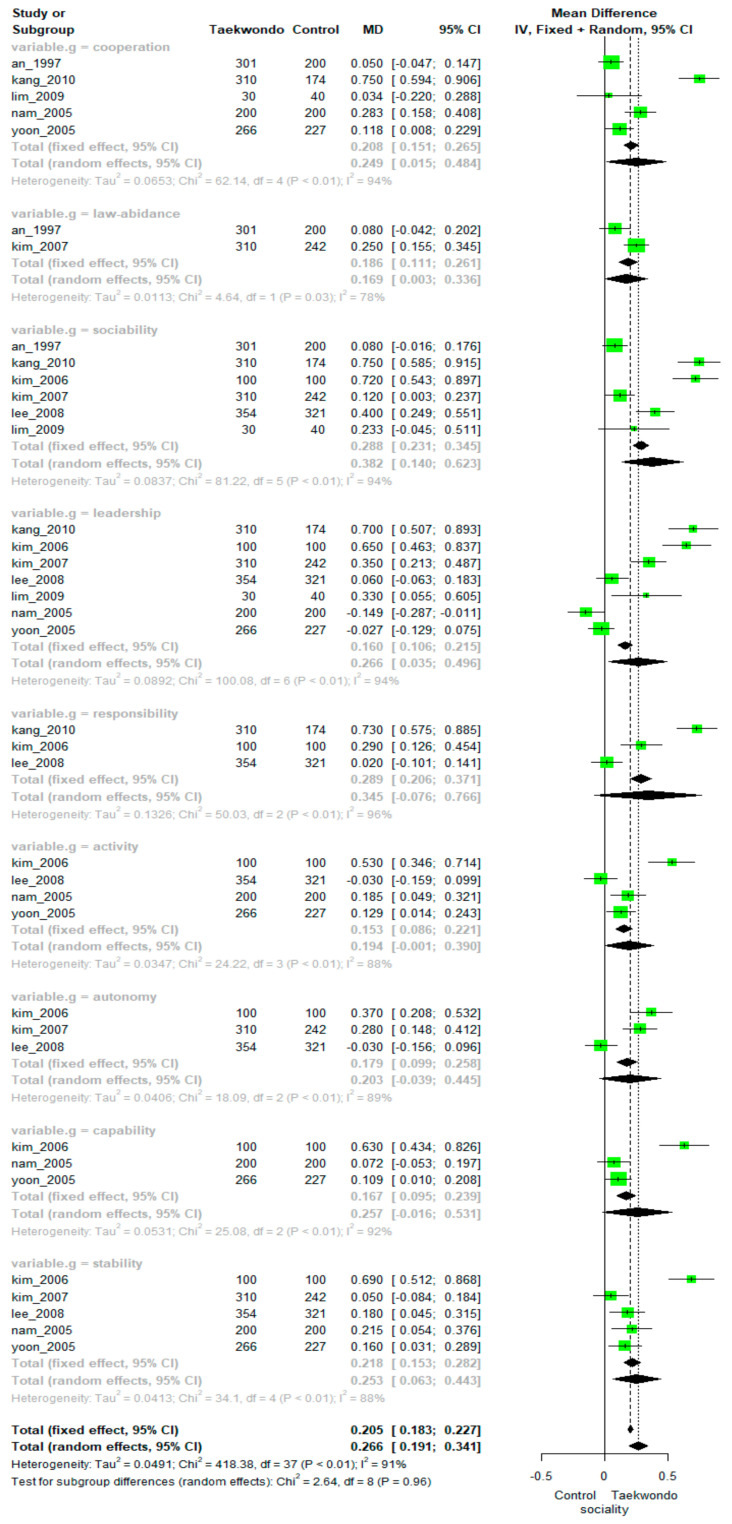
Forest plot of Taekwondo effects on sociality.

Pooling data from eight studies showed a significant positive effect of Taekwondo training on sociality (MD = 0.266; 95% CI, 0.191–0.341). There was high heterogeneity among the studies (*p* < 0.01, I^2^ = 91%). The differences between the subfactors were not statistically significant (*p =* 0.96). The overall effect size depending on the subfactors of sociality (leadership, sociability, stability, capability, autonomy, law-abidance, responsibility, cooperation, and activity) is presented in Figure 2. 

Specifically, meta-analyses found significant positive effects of Taekwondo training on sociality in the following subfactors: cooperation (MD = 0.249, 95% CI: 0.015 to 0.484; heterogeneity: I^2^ = 94%, *p* < 0.01), law compliance (MD = 0.169, 78%CI: 0.060 to 0.403; heterogeneity: I^2^ = 78%*, p* = 0.03), leadership (MD = 0.266, 95% CI: 0.035 to 0.496; heterogeneity: I^2^ = 94%, *p* < 0.01), responsibility (MD = 0.345, 95% CI: −0.076 to 0.766; heterogeneity: I^2^ = 96%, *p* < 0.01), sociability (MD = 0.382, 95% CI: 0.140 to 0.623; heterogeneity: I^2^ = 94%, *p* < 0.01), and stability (MD = 0.253, 95% CI: 0.063 to 0.443; heterogeneity: I^2^ = 88%, *p* < 0.01).

However, there were no significant differences between the Taekwondo training groups and controls in the following subfactors of sociality: activity (MD = 0.194, 95% CI: −0.001 to 0.390; heterogeneity: I^2^ = 88%, *p* < 0.01), autonomy (MD = 0.203, 95% CI: −0.039 to 0.445; heterogeneity: I^2^ = 89%, *p* < 0.01), and capability (MD = 0.257, 95% CI: −0.016 to 0.531; heterogeneity: I^2^ = 92%, *p* < 0.01).

#### 3.2.2. Character

**Figure 3:**
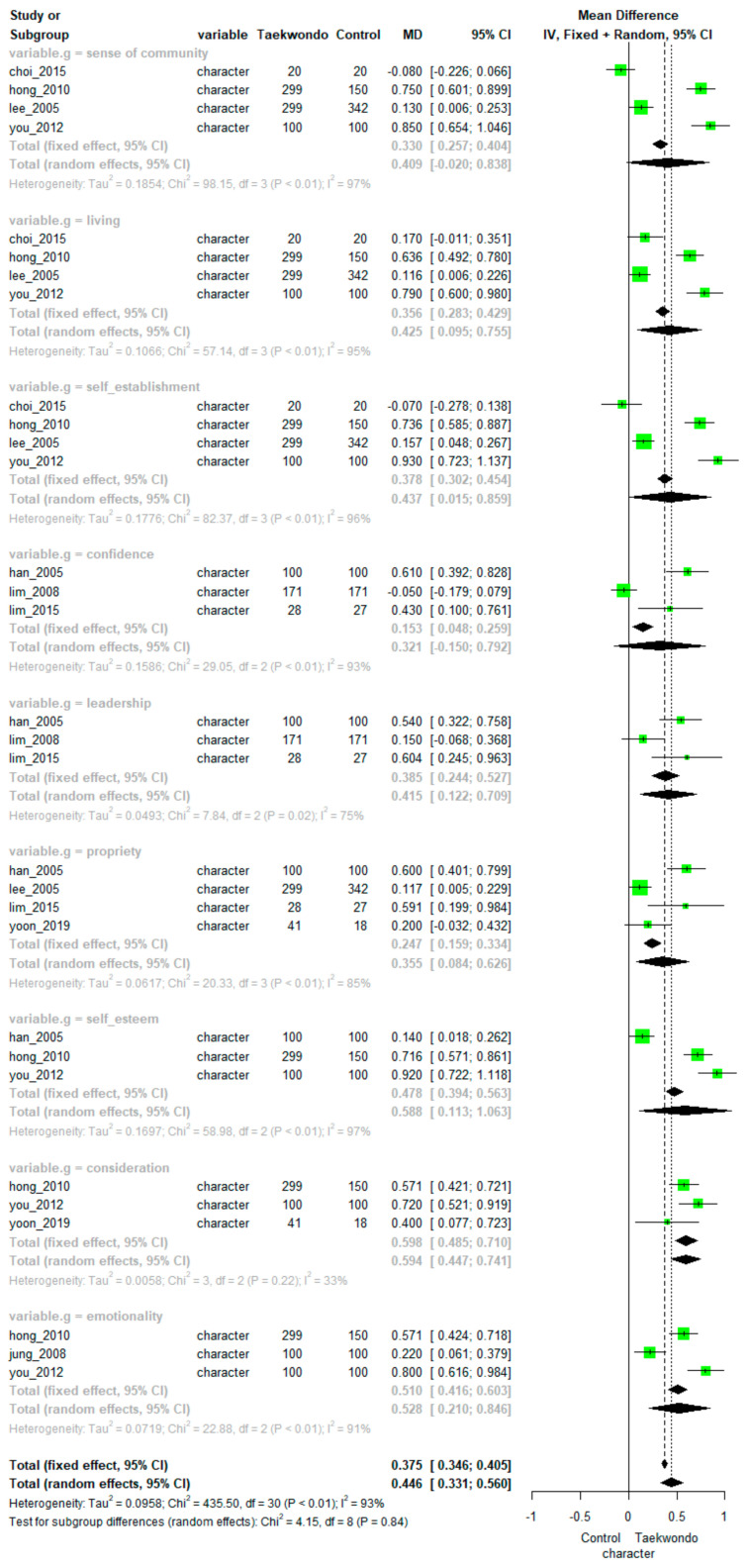
Forest plot of Taekwondo effects on character.

Pooling data from nine studies showed a significant positive effect of Taekwondo training on character (MD = 0.446, 95% CI: 0.331 to 0.560). There was high heterogeneity among the studies (*p* < 0.01, I^2^ = 93%). The differences between the subfactors were not statistically significant (*p =* 0.84). The overall effect size depending on the subfactors of character (sense of community, confidence, consideration, emotionality, leadership, living, propriety, self-establishment, and self-esteem) is presented in Figure 3.

Specifically, meta-analyses found significant positive effects of Taekwondo training on character in the following subfactors: sense of community (MD = 0.520, 95% CI: 0.190 to 0.850; heterogeneity: I^2^ = 95%, *p* < 0.01), consideration (MD = 0.544, 95% CI: 0.344 to 0.744; heterogeneity: I^2^ = 62%, *p* = 0.07), emotionality (MD = 0.506, 95% CI: 0.201 to 0.810; heterogeneity: I^2^ = 91%, *p* < 0.01), leadership (MD = 0.393, 95% CI: 0.130 to 0.656; heterogeneity: I^2^ = 72%, *p* = 0.03), propriety (MD = 0.316, 95% CI: 0.047 to 0.585; heterogeneity: I^2^ = 84%, *p* < 0.01), living (MD = 0.402, 95% CI: 0.083 to 0.721; heterogeneity: I^2^ = 95%, *p* < 0.01), self-establishment (MD = 0.512, 95% CI: 0.184 to 0.840; heterogeneity: I^2^ = 95%, *p* < 0.01), and self-esteem (MD = 0.561, 95% CI: 0.118 to 1.005; heterogeneity: I^2^ = 96%, *p* < 0.01).

However, there was no significant difference between the Taekwondo training groups and controls in the confidence subfactor (MD = 0.324, 95% CI: −0.150 to 0.792, *p* < 0.01; heterogeneity: I^2^ = 93%).

#### 3.2.3. Etiquette

**Figure 4:**
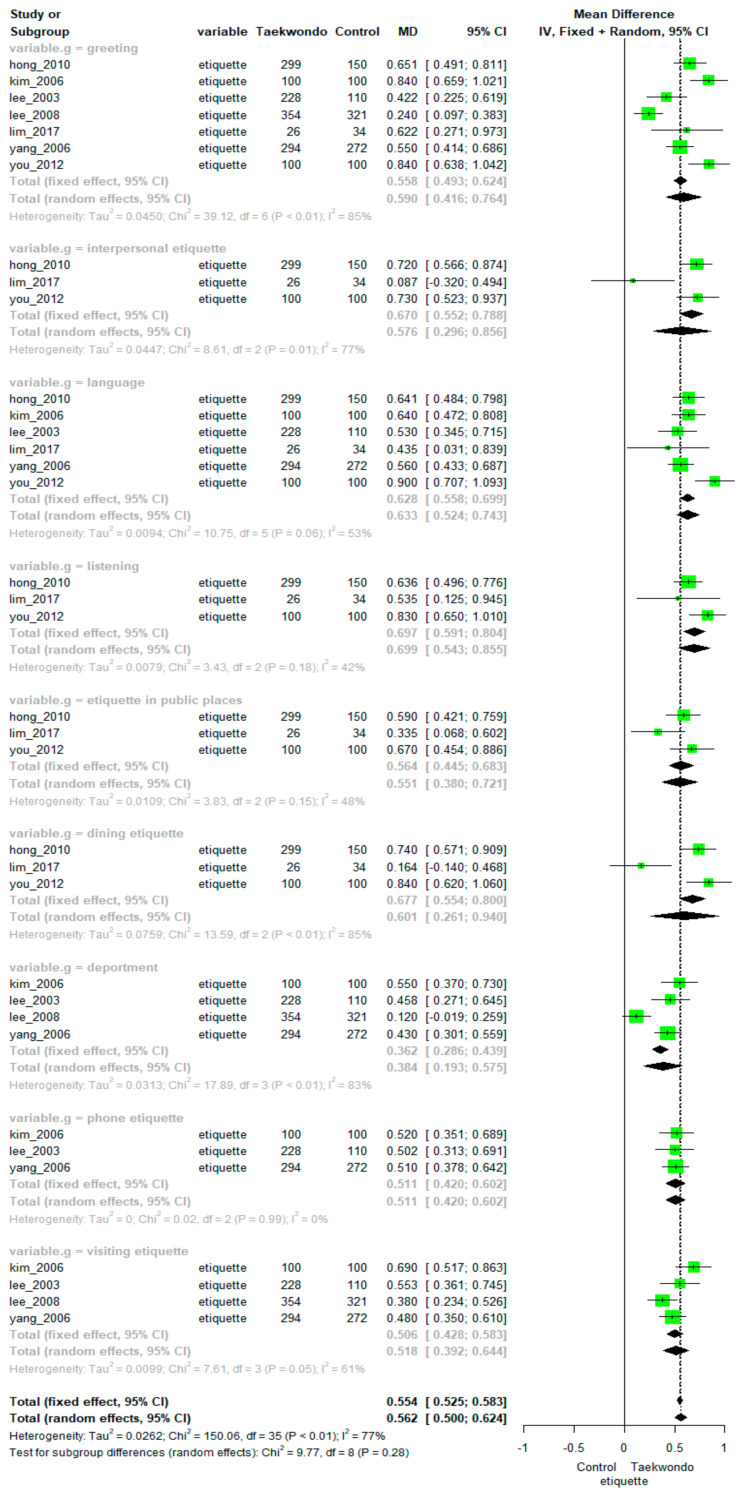
Forest plot of Taekwondo’s effects on etiquette.

Pooling data from seven studies showed a significant positive effect of Taekwondo training on etiquette (MD = 0.562, 95% CI: 0.500–0.624). There was high heterogeneity among the studies (*p* < 0.01, I^2^ = 77%). The differences between the subfactors were not statistically significant (*p =* 0.28). The overall effect size depending on the subfactors of etiquette (deportment, greeting, interpersonal etiquette, language, listening, phone etiquette, etiquette in public places, dining etiquette, and visiting etiquette) is presented in Figure 4.

Specifically, meta-analyses found significant positive effect of Taekwondo training on etiquette in all the subfactors: deportment (MD = 0.384, 95% CI: 0.193 to 0.575; heterogeneity: I^2^ = 83%, *p* < 0.01), greeting (MD = 0.590, 95% CI: 0.416 to 0.764; heterogeneity: I^2^ = 85% *p* < 0.01), interpersonal etiquette (MD = 0.577, 95% CI: 0.296 to 0.856; heterogeneity: I^2^ = 77% *p* = 0.01), language (MD = 0.633, 95% CI: 0.524 to 0.743; heterogeneity: I^2^ = 53%, *p* = 0.06), listening (MD = 0.699, 95% CI: 0.543 to 0.855; heterogeneity: I^2^ = 42%, *p* = 0.18), phone etiquette (MD = 0.511, 95% CI: 0.420 to 0.602; heterogeneity: I^2^ = 0%, *p* = 0.99), etiquette in public places (MD = 0.551, 95% CI: 0.380 to 0.721; heterogeneity: I^2^ = 48%, *p* = 0.15), dining etiquette (MD = 0.601, 95% CI: 0.261 to 0.940; heterogeneity: I^2^ = 85%, *p* < 0.01), and visiting etiquette (MD = 0.518, 95% CI: 0.392 to 0.644; heterogeneity: I^2^ = 61%, *p* = 0.05.

#### 3.2.4. School Life Adjustment

**Figure 5:**
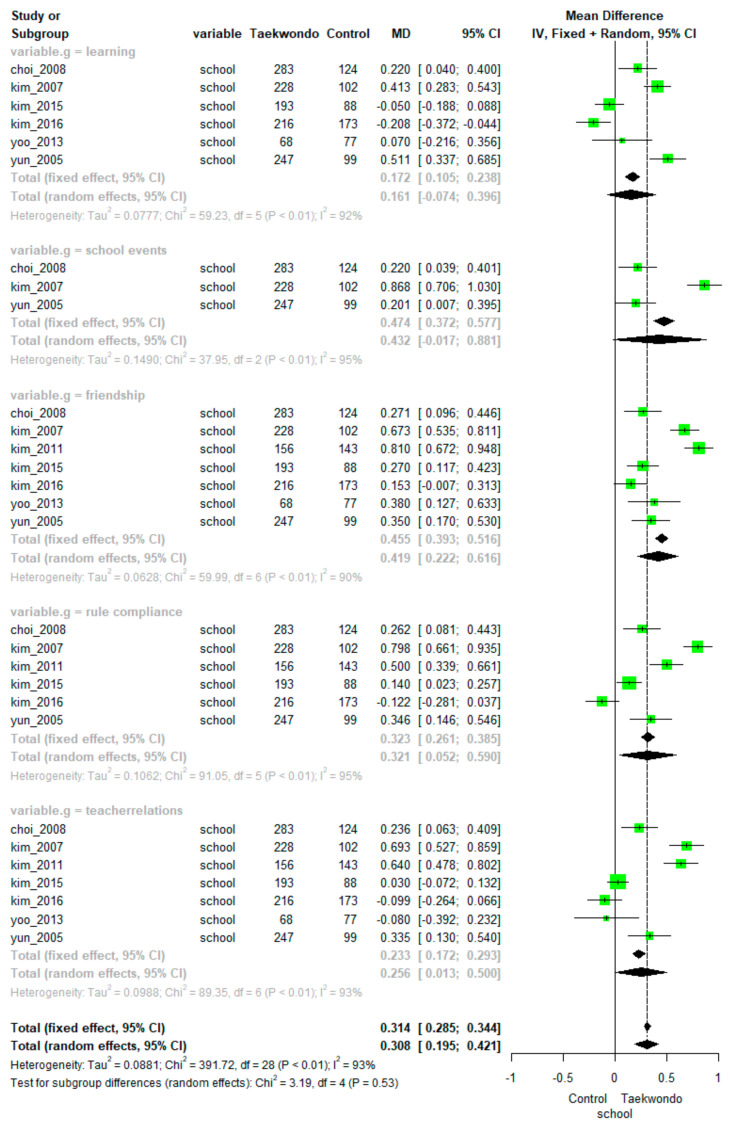
Forest plot of Taekwondo effects on school life adjustment.

Pooling data from seven studies showed a significant positive effect of Taekwondo training on school life adjustment (MD = 0.308, 95% CI: 0.195 to 0.421). There was high heterogeneity among the studies (*p* < 0.01, I^2^ = 94%). The differences between the subfactors were not statistically significant (*p =* 0.53). The overall effect size depending on the subfactors of school life adjustment (learning, school events, friendship, rule compliance, and teacher relations) is presented in Figure 5.

Meta-analyses found a significant positive effect of Taekwondo training on school life adjustment in the subfactors of learning (MD = 0.161, 95% CI: -0.074 to 0.396; heterogeneity: I^2^ = 92%, *p* < 0.01), friendship (MD = 0.419, 95% CI: 0.222 to 0.616; heterogeneity: I^2^ = 90%, *p* < 0.01), rule compliance (MD = 0.321, 95% CI: 0.052 to 0.590; heterogeneity: I^2^ = 95%, *p* < 0.01), and teacher relations (MD = 0.256, 95% CI: 0.013 to 0.500; heterogeneity: I^2^ = 93%, *p* < 0.01).

However, there was no significant difference between the Taekwondo training groups and controls in the school events subfactor (MD = 0.324, 95% CI: −0.150 to 0.792, *p* < 0.01; heterogeneity: I^2^ = 96%).

### 3.3. Moderator Analysis

We performed a meta-ANOVA to detect any moderation effects of the categorical variables: study type (cross-sectional or prospective cohort), sex (male proportion), age (elementary, middle, high, or adult), study quality (good, fair, or poor), publication type (thesis or journal article), and meta-regression for the continuous variable (i.e., sample size) on sociality, character, etiquette, and school life adjustment separately.

The meta-ANOVA yielded a significant moderation effect of sex (*p* < 0.001) in sociality, while the moderation effect of age was found to be significant (*p* < 0.001) in character. Furthermore, etiquette showed significant moderation effects of study type (*p* = 0.026), age (*p* < 0.001), publication type (*p* = 0.022), and sample size (*p* = 0.018). In school life adjustment, sex (*p* = 0.027), age (*p* = 0.003), and study quality (*p* = 0.038) emerged as significant moderators.

### 3.4. Publication Bias and Sensitivity Analyses

#### 3.4.1. Sociality

In the analysis of publication bias, the *p*-value was found to be 0.001 in both Egger’s test and Begg’s test. The contour funnel plot showed that 29 studies had a *p*-value of less than 0.05. Sensitivity analysis for publication bias using the trim-and-fill method revealed the adjusted overall effect size (MD = 0.138, 95% CI: 0.055 to 0.220), suggesting a significant improvement in sociality in the Taekwondo training group. There was significant heterogeneity among the included studies (I^2^ = 93.9%), *p* < 0.01).

#### 3.4.2. Character

As for publication bias, the *p*-value was found to be 0.025 and 0.114 in Egger’s test and Begg’s test, respectively. There were 26 studies with a *p*-value of less than 0.05. We performed sensitivity analysis for publication bias using the trim-and-fill method, which revealed the adjusted overall effect size (MD = 0.2659, 95% CI: 0.152 to 0.378), suggesting a significant character improvement in the Taekwondo training group. There was significant heterogeneity among the included studies (I^2^ = 94.8%). *p* < 0.01).

#### 3.4.3. Etiquette

In the analysis of publication bias, the *p*-values were 0.628 and 0.549 in Egger’s test and Begg’s test, respectively. The contour funnel plot showed that 33 studies had a *p*-value of less than 0.05. Sensitivity analysis for publication bias using the trim-and-fill method revealed the adjusted overall effect size (MD = 0.534, 95% CI: 0.472–0.596), suggesting a significant improvement in etiquette in the Taekwondo training group. There was significant heterogeneity among the included studies (I^2^ = 78.4%), *p* < 0.01).

#### 3.4.4. School Life Adjustment

For publication bias, the *p*-value was found to be 0.912 and 0.822 in Egger’s test and Begg’s test, respectively. There were 23 studies with a *p*-value of less than 0.05. Since there was no additional study, the adjusted overall effect size yielded by a sensitivity analysis was the same as the existing overall effect size.

## 4. Discussion

This meta-analysis was conducted to examine Taekwondo training’s effect on psychosocial variables such as sociality, character, etiquette, and school life adjustment. In this review of 28 studies, including cross-sectional and intervention studies, the results indicated that Taekwondo training had small to medium effects on psychosocial factors. The interpretation of the results, implications, and limitations is discussed separately by the variables. 

### 4.1. Sociality

Our analysis revealed that Taekwondo had a positive effect on sociality in the subfactors of cooperation, law-abidance, leadership, responsibility, sociability, and stability, with a small overall effect size ranging between 0.203 and 0.396. Among the eight studies, seven were master’s theses with a cross-sectional design. Only one peer-reviewed study examined the effect of Taekwondo training by employing a 24-week intervention [3]. As demonstrated in Table 1, six of the included studies targeted elementary school students, mostly upper graders, while two targeted high school students. It is interesting to note that all of the cross-sectional studies included in our meta-analysis reported improvement in social characteristics in general, although differences existed among the subfactors of sociality assessed in each individual study. However, an intervention study by Tae-Hee Lim [3] found that the 24-week Taekwondo program did not result in meaningful improvement in sociality compared to controls. Considering that the results from the cross-sectional studies consistently reported a positive correlation between the sociality outcomes and the years of Taekwondo training [22,23,25], future studies should adopt intervention programs for an extended period of time to examine the cause-and-effect relationship between Taekwondo and social development.

A meta-analysis of studies examining Taekwondo’s effect on sociality revealed that Taekwondo training in the long term might be effective for an individual’s social development. However, due to the relatively small effect size and the study type (i.e., cross-sectional) of the literature synthesized in this meta-analysis, caution needs to be taken to avoid overestimating the link between them. Furthermore, Taekwondo’s overall effect on sociality subfactors such as activity, autonomy, and capability were not significant.

We found that, although some studies reported significant correlations between the sociality outcomes of Taekwondo training students and their demographic characteristics, such as the parents’ education and income level [22,25], none of these studies further assessed the effects of Taekwondo on the linkage between socioeconomic status and social development. As underserved groups are often known to be more sensitive to exercise effects, having greater room for improvement [34], future studies are encouraged to explore whether Taekwondo training’s effect on social development differs depending on socioeconomic factors.

### 4.2. Character

The results of this meta-analysis indicated that Taekwondo training had a positive impact on character in the subfactors of sense of community, consideration, emotionality, leadership, propriety, living, self-establishment, and self-esteem, with small to medium overall effect sizes ranging between 0.311 and 0.560. Among the nine studies, six were cross-sectional and three were intervention studies. Since the synthesized studies included age groups from preschoolers and elementary school students to high school students and female college students, it appears that Taekwondo training’s beneficial effect on character development can be applied to practitioners of various ages. In Yoon’s study [35], high school students who participated in morning Taekwondo sessions for nine months exhibited greater improvement in character assessment relative to controls. Similarly, in a cross-sectional study by You [34], which compared character elements of female undergraduates who practiced Taekwondo with those who did not, the Taekwondo practitioners were found to have better emotional stability, self-respect, consideration, sense of community, and self-establishment. Consistently, an intervention study with preschool children [10] and the remaining six studies with elementary school students reported greater character enhancement in the Taekwondo training group than in the non-training group.

The principle that Taekwondo brings about positive character development in trainees, especially children, is closely related to the solemn and educational atmosphere of Taekwondo academies and training program content that is intentionally planned and delivered by the Sabum-nim (i.e., master instructor) [31]. As part of the training, they usually include elements of character education such as greeting, etiquette, respect, consideration, rules of conduct, etc. For this reason, regular participation in Taekwondo in the long run not only contributes to personal growth but also to relationships with others, as internalization of these lessons can be transferred to daily life in school and at home [10]. A trainee who has earned a black belt after long and difficult training feels a strong sense of achievement, and these juniors are often given the role of instructor assistants during training, helping other trainees with Poomsae or leading Gihap (i.e., a shout of concentration). These experiences enhance the trainees’ leadership and consideration of others, as well as fostering self-esteem and self-efficacy [10,29].

One limitation we observed in the process of this research is that the three variables (i.e., sociality, character, and etiquette) are so closely interconnected that the categorization or conceptualization of the variables varied among studies. For example, Lim [13] divided character factors into individual (etiquette and self-confidence) and interpersonal relationships (leadership). In addition, some studies have overlapping subfactors in different variables. For example, Kang [22] included leadership as a subfactor of sociality, whereas Han [29] viewed leadership as a subfactor of character. Although we performed this meta-analysis according to the categorization intended by the original researchers, future studies will need to better control the effects of the variance in research methods and conceptualization.

### 4.3. Etiquette

Our analysis found that Taekwondo training had a positive effect on etiquette in all of the tested subfactors (deportment, greeting, interpersonal etiquette, language, listening, phone etiquette, etiquette in public places, dining etiquette, and visiting etiquette), with a medium overall effect size ranging between 0.50 and 0.60. Except for the study by Lim [30], which investigated the effect of a six-month Taekwondo intervention on etiquette in elementary school students, the remaining six studies were cross-sectional studies that compared the self-assessed etiquette of existing Taekwondo trainees and non-trainees. The results showed that the Taekwondo training groups had better etiquette than the non-training groups. Furthermore, Taekwondo’s positive impact on etiquette was found to be greater as the training experience increased [23,37].

Proper etiquette is an important component in Taekwondo, as reflected in the saying “Taekwondo begins with manners and ends with manners.” Taekwondo instructors continuously educate the trainees to be polite, behave properly, and show respect to others, which becomes a habit expressed in the trainee’s life at home and at school, and these basic behaviors develop into good manners. Trulson [45] argued that the traditional form of Taekwondo, which emphasizes martial arts philosophy, is effective for positive youth development. Previous studies also support that the traditional Taekwondo as a martial art has greater psychosocial benefit compared to modern Taekwondo as a sport [46,47]. Considering that the purpose of traditional Taekwondo is not about beating the opponent or winning the match, but rather to improve self-control, it is understandable that training programs including education in the mental and philosophical aspects of Taekwondo might be more useful for psychosocial development than the game-oriented style of Taekwondo training. The differential training effects of these two distinct approaches should be explored in depth in future studies.

### 4.4. School Life Adjustment

Our meta-analysis revealed that Taekwondo had a positive effect on school life adjustment in the subfactors of learning, friendship, rule compliance, and teacher relations, with small overall effect sizes ranging between 0.195 and 0.421. The seven included studies were all cross-sectional. Six of these studies were of elementary school students, and the other was of high school students. The synthesis of these studies yielded significant differences in school life adjustment depending on Taekwondo participation. Students who took part in Taekwondo training were shown to have higher scores on the measures of school life adjustment compared to non-trainees. These results imply that Taekwondo training taking place in a peer group may facilitate socialization of the student trainees, which ultimately contributes to school life where they spend a great deal of time with peers. 

Furthermore, studies have reported that individuals who train in Taekwondo have better self-control compared to non-trainees, with certified trainees demonstrating even better self-control (reference). In the study by Choi [9], children with better self-control in attention, composure, rule compliance, and interpersonal relationships showed better adaptation to school life. These findings suggest that enhanced self-control through Taekwondo training is closely related to school life adjustment.

It appears that trainees have opportunities to learn attitudes and values such as sportsmanship, rule-observance, self-discipline, role sharing, and cooperation during Taekwondo practice, which are highly effective and important skills for adapting to the school community and establishing a satisfactory life at school. However, since the present research only included cross-sectional studies, caution is required in interpreting these results as a direct effect of Taekwondo training. It would be more appropriate to view Taekwondo’s effect on school life adjustment as a significant relationship, rather than causality. Thus, future research will need to investigate its effect on school life adjustment using randomized controlled trials, with varying lengths and trainees’ socioeconomic characteristics taken into consideration.

### 4.5. Limitations

This meta-analysis has several limitations. First, differences between studies exist in the lengths of Taekwondo training or intervention, which could lead to heterogeneity across the included studies. Second, among the 28 studies included, only four used intervention designs, while the rest were cross-sectional. Therefore, to confirm causality between Taekwondo training and psychosocial benefits, more interventional research should be conducted due to the paucity of current data published on this issue. Third, excluding studies conducted and published in countries other than Korea might have biased our findings. However, as Korea is Taekwondo’s place of origin, it is widely distributed and actively studied. In addition, limiting the inclusion criteria to one country reduced the potential influence of cultural and geographical differences.

Furthermore, researchers used self-report questionnaires to assess psychosocial factors of the study participants in the majority of the included studies, asking them to evaluate their own status. Thus, data can easily be affected by the loss or distortion of memory and the truthfulness of the responses. Therefore, future studies would be more convincing if they adopt more objective and direct measures for investigating the psychosocial effects of Taekwondo. Finally, due to the selected studies and the inevitable heterogeneity among them, the pooled results might include the biases of individual studies as well as new sources of bias, which is a common limitation of meta-analysis. Therefore, future meta-analyses on this topic should integrate more prospective cohort studies or RCTs to confirm the findings of this research.

## 5. Conclusions

In summary, this meta-analysis indicates that Taekwondo training has positive effects on psychosocial factors such as sociality, character, etiquette, and school life adjustment. Specifically, Taekwondo trainees exhibited significantly higher self-assessed scores on cooperation, law-abidance, leadership, responsibility, sociability, and stability among the subfactors of sociality. In the character subfactor, Taekwondo trainees were found to have a higher sense of community, consideration, emotionality, leadership, propriety, living, self-establishment, and self-esteem. In terms of etiquette, Taekwondo trainees had better qualities in terms of deportment, greeting, interpersonal etiquette, language, listening, phone etiquette, etiquette in public places, dining etiquette, and visiting etiquette. Finally, the improved psychosocial characteristics seem to be associated with better school life adjustment, as evidenced by higher scores on learning, friendship, rule compliance, and teacher relations in Taekwondo training students relative to non-trainees. However, several concerns, including inherent biases in the sample type, outcome measurements, and intervention settings included in this meta-analysis may limit definitive conclusions. Future studies are encouraged to conduct well-designed RCTs and multiple levels of Taekwondo intervention to validate the findings of the current analysis.

## Figures and Tables

**Table 1:** Characteristics of included studies.

1. Sociality					
Author, Year	No. Participants (TKD, Control)	% Male	Age Group	Covariates	Study Type (Quality Score)
Kang, 2010 [22]	484 (310, 174)	64.26%	elementary school	sex, grade, period, time, frequency	cross-section (9)
Kim, 2006 [23]	200 (100, 100)	50.00%	high school	sex, parent’s education, standard of living, health, period	cross-section (9)
Kim, 2007 [24]	330 (228, 102)	61.21%	elementary school	sex, age, period	cross-section (9)
Nam, 2005 [25]	400 (200, 200)	75.00%	elementary school	parent’s education, standard of living, period	cross-section (9)
An, 1997 [26]	501 (301, 200)	58.00%	elementary school	sex, grade, parent’s education, period	cross-section (9)
Yoon, 2005 [27]	493 (266, 227)	71.40%	elementary school	sex, grade,	cross-section (9)
Lee, 2008 [28]	675 (354, 321)	unknown	high school	grade, academic achievement, period, frequency, time	cross-section (7)
Lim,2009 [3]	70 (30, 40)	unknown	elementary school	unknown	Intervention (8) 24-week
**2. Character**					
**Author, Year**	**No. Participants (TKD, Control)**	**% Male**	**Age Group**	**Covariates**	**Study Type (Quality Score)**
Choi, 2015 [10]	40 (20, 20)	14.48%	preschool	-	Intervention (9) 8-week
Han, 2005 [29]	200 (100, 100)	50%	elementary school	sex, grade	cross-section (9)
Hong, 2010 [30]	449 (299, 150)	48.55%	elementary school	sex, grade, period, time	cross-section (9)
Jung, 2007 [31]	200 (100, 100)	50%	elementary school	sex, grade, parent’s job, career, period, frequency, a single-parent family, number of siblings	cross-section (9)
Lee,2005 [32]	642 (399, 342)	53.20%	elementary school	sex, grade, training place, period, frequency, time	cross-section (7)
Lim, 2008 [33]	342 (171, 171)	65.50%	elementary school	sex, grade, academic achievement, career, tutoring, standard of living	cross-section (8)
Lim, 2015 [13]	55 (28, 27)	unknown	elementary school	-	intervention (9) 12-week
You, 2012 [34]	200 (100, 100)	0%	college	grade, frequency	cross-section (7)
Yoon, 2019 [35]	59 (41, 18)	6.78%	high school	Sex, grade, certification	intervention (9) 9-month
**3. Etiquette**					
**Author, Year**	**No. Participants (TKD, Control)**	**% Male**	**Age Group**	**Covariates**	**Study Type (Quality Score)**
Kim, 2006 [23]	200 (100, 100)	50.00%	high school	sex, parent’s education, standard of living, health, period	cross-section (9)
Lee, 2003 [36]	338 (228, 110)	74.56%	elementary school	sex, grade, parent’s education	cross-section (9)
Lee, 2008 [28]	675 (354, 321)	unknown	high school	grade, academic achievement, period, frequency, time	cross-section (7)
Yang, 2006 [37]	566 (294, 272)	59.36%	elementary school	sex, age, standard of living, religion, parent’s education	cross-section (7)
Hong, 2010 [30]	449 (299, 150)	48.55%	elementary school	sex, grade, period, time	cross-section (9)
Lim, 2017 [38]	60 (26, 34)	unknown	elementary school	-	intervention (9) 6-month
You, 2012 [34]	200 (100, 100)	0%	college	grade, frequency	cross-section (7)
**4. School Life Adjustment**					
**Author, Year**	**No. Participants (TKD, Control)**	**% Male**	**Age Group**	**Covariates**	**Study Type (Quality Score)**
Choi, 2008 [9]	407 (283, 124)	65.60%	elementary school	sex, age, parent’s education, period, career	cross-section (9)
Kim, 2007 [39]	552 (310, 242)	62.86%	elementary school	sex, grade	cross-section (7)
Kim, 2011 [40]	299 (156, 143)	100%	elementary, middle school	grade	cross-section (8)
Kim, 2015 [41]	281 (193, 88)	56.94%	elementary school	sex, grade	cross-section (8)
Kim, 2016 [42]	389 (216, 173)	46.79%	middle school	sex, grade, residence, academic achievement	cross-section (8)
Yoo, 2013 [43]	145 (68, 77)	51.72%	elementary school	sex, grade	cross-section (5)
Yoon, 2005 [44]	360 (240, 120)	60.12%	elementary school	sex, age, standard of living, religion, parent’s education	cross-section (9)
